# Genome-Wide Identification and Functional Characterization of the BAHD Acyltransferase Gene Family in *Brassica napus* L.

**DOI:** 10.3390/plants14142183

**Published:** 2025-07-15

**Authors:** Yuanyuan Liu, Xingzhi Wei, Yiwei Liu, Yunshan Tang, Shulin Shen, Jie Xu, Lulu Chen, Cunmin Qu, Huiyan Zhao, Hai Du, Huafang Wan, Nengwen Yin, Ti Zhang

**Affiliations:** 1 Integrative Science Center of Germplasm Creation in Western China (Chongqing) Science City, College of Agronomy and Biotechnology, Southwest University, Chongqing 400715, China; lyy18865550726@163.com (Y.L.); weixingzhi031026@163.com (X.W.); liuyiwei1942@email.swu.edu.cn (Y.L.); tys1998@email.swu.edu.cn (Y.T.); ssl7159@email.swu.edu.cn (S.S.); 15723480213@163.com (J.X.); zxhcsgd1023@email.swu.edu.cn (L.C.); drqucunmin@swu.edu.cn (C.Q.); zhaohuiyan@swu.edu.cn (H.Z.); haidu81@126.com (H.D.); wanhua05@swu.edu.cn (H.W.); nwyin80@swu.edu.cn (N.Y.); 2 Academy of Agricultural Sciences, Southwest University, Chongqing 400715, China; 3 Engineering Research Center of South Upland Agriculture, Ministry of Education, Chongqing 400715, China

**Keywords:** BAHD acyltransferase, *Brassica napus* L., acylation, stress resistance

## Abstract

The BAHD acyltransferase family plays a critical role in plant secondary metabolism by catalyzing acyl transfer reactions that are essential for synthesizing metabolites involved in environmental adaptation. However, systematic investigation of this superfamily in *Brassica napus* has not been reported. In this study, 158 *BnaBAHD* genes were identified by comprehensive analyses of evolutionary relationships, motif structures, chromosomal distribution, gene collinearity, and selection pressures, and these genes were phylogenetically classified into five clades harboring conserved catalytic domains (HXXXD and DFGWG). Transient overexpression combined with metabolomic profiling demonstrated that two homologous seed-specific Clade V members, *BnaBAHD040* and *BnaBAHD120*, which exhibited elevated expression during late seed development, significantly enhanced the accumulation of acylated metabolites contributing to biotic/abiotic stress resistance. This study provides the first experimental validation of the catalytic functions of BAHD enzymes in *B. napus*, establishing a theoretical foundation for leveraging this gene family in genetic improvement to develop novel rapeseed cultivars with enhanced stress tolerance and yield.

## 1. Introduction

Plants synthesize a vast array of secondary metabolites during their growth and development. The diversity and complexity of these compounds are influenced by various biochemical modifications, including oxidation/reduction, methylation, glycosylation, acylation, phosphorylation, and others [1,2]. Among these, acylation is a prevalent and critical modification process that significantly impacts the structural diversity, functional properties, and bioactivities of plant secondary metabolites [3]. The BAHD acyltransferase family, a key player in acylation, primarily utilizes activated coenzyme A (CoA) thioesters as acyl donors and the hydroxyl or amine groups of acceptor molecules to catalyze acylation reactions [1]. This process leads to the formation of diverse acylated compounds, such as esters, anthocyanins, terpenoids, lignin monomers, and cutins, which are essential for plant metabolism and function [4].

The BAHD acyltransferase family derives its name from the initials of the first four enzymes identified within this superfamily, each of which exhibits distinct biochemical properties [5]. These include the following: Benzylalcohol O-acetyltransferase (BEAT) from *Clarkia breweri*, which participates in the biosynthesis of volatile ester compounds [6]; Anthocyanin O-hydroxycinnamoyltransferase (AHCT) from *Gentiana triflora*, responsible for synthesizing acylated anthocyanins [7]; Anthranilate N-hydroxycinnamoyl/benzoyltransferase (HCBT) from *Dianthus caryophyllus*, which facilitates the initial committed reaction in the biosynthesis of a class of phytoalexins known as anthramides [8]; and Deacetylvindoline 4-O-acetyltransferase (DAT) from *Catharanthus roseus*, which acts as the terminal enzyme in the biosynthesis of the alkaloid vindoline [6]. Structurally, most members of the BAHD family are monomeric enzymes with molecular weights ranging from 48 to 55 kDa, typically encoding approximately 445 amino acids [9]. While the majority of these enzymes are localized in the cytoplasm, some, such as MtMaT1 in *Medicago truncatula* and tobacco leaf cells, are also found in the nucleus [10].

BAHD acyltransferases catalyze acylation through a conserved two-step mechanism involving two signature motifs: the CoA-binding DFGWG motif and the catalytic HXXXD motif [1,11]. During the activation phase, the carboxyl group (COOH) of the acyl donor undergoes CoA esterification to form an activated acyl-CoA intermediate [11,12]. This step is stabilized by the DFGWG motif, which facilitates substrate binding through interactions with the CoA moiety [11,12]. The subsequent transacylation step is mediated by the HXXXD motif located near the catalytic core [13]. This motif orchestrates the deprotonation of hydroxyl or amine groups on acceptor molecules, enabling nucleophilic attack on the acyl-CoA carbonyl carbon to complete *O*-acylation or *N*-acylation [12]. In contrast to the nearly complete conservation of the HXXXD motif across BAHD family members, the DFGWG motif exhibits relatively larger variation among the identified gene models [14]. Take genes in poplar, for example: the DFGWG motif is frequently transformed into DFGFG, DFGWA, DFGWK, NFGWG, and so on; moreover, PtACT66 exhibits a conspicuous absence of DFGWG-like motifs [14].

The BAHD acyltransferase family has emerged as a key research focus in plants due to its critical role in plant metabolism. Members of BAHD enzyme family have been identified in numerous species, including *Taxus mairei*, *Camellia sinensis*, *Hordeum vulgare*, *Populus*, *Arabidopsis thaliana*, *Pyrus bretschneideri*, *Lavandula angustifolia*, which has significantly advanced our understanding of this enzyme family [4,14,15,16,17,18,19]. As a multifunctional enzyme family, BAHD members exhibit diverse biological roles in stress adaptation and developmental regulation. For instance, in *Arabidopsis*, EPS1, a BAHD acyltransferase family protein, collaborates with PBS3 to convert isochorismate into salicylic acid (SA) via a two-step metabolic pathway, enhancing pathogen resistance by promoting SA accumulation [20]. Another BAHD member, DCR, is essential for cutin polymer formation and modulates tolerance to salinity, osmotic stress, and water deprivation [21]. In fruits such as strawberry, apple, and peach, the expression of BAHD acyltransferase family members (e.g., *AAT*) is upregulated during ripening and postharvest stages, mediating ester biosynthesis to directly shape fruit flavor profiles [22,23,24]. Collectively, as a key gene family in plant secondary metabolism that catalyzes acyl transfer reactions, *BAHDs* mediate structural diversification (e.g., esterification and amidation) of small-molecule compounds, thereby regulating critical biological processes such as plant defense, signal transduction, and environmental adaptation [1,16,25,26,27].

As the world’s second-largest oilseed crop, rapeseed holds critical agricultural importance, contributing 13–16% of global edible oil production while demonstrating substantial economic value and developmental prospects [28,29]. It is an allopolyploid crop that originated from the hybridization of *Brassica rapa* and *Brassica oleracea*, followed by natural chromosome doubling [30]. At present, there is a lack of systematic research on the BAHD gene family in *B. napus*, and its genome-wide analysis has not been reported yet. Therefore, systematic identification of BAHD gene family members and comprehensive characterization of their expression patterns in *B. napus* will provide a foundational framework for elucidating the biological functions of *BnaBAHDs*, including their potential roles in stress responses, developmental processes, and metabolic regulation, thereby advancing functional genomics research in *B. napus*.

In the current study, we identified 158 *BnaBAHD* genes through genome-wide analysis and comprehensively characterized their evolutionary relationships, motif structures, chromosomal distributions, gene collinearity, and selection pressures, representing the first comprehensive study of this gene family in *B. napus*. Additionally, heterologous expression and catalytic function analysis provided a theoretical basis for further research on *BAHD* genes in *B. napus*.

## 2. Results

### 2.1. Identification and Characterization of BAHD Family Genes in Arabidopsis and Three Brassica Species

We identified 395 BAHD family members across three *Brassica* species and *Arabidopsis*, including 91 *BraBAHDs* in *B. rapa*, 83 *BolBAHDs* in *B. oleracea*, and 158 *BnaBAHDs* in *B. napus*, along with 63 candidate genes in *Arabidopsis*. Eight candidate genes were excluded due to incomplete structural domains, ensuring all retained members contained at least one of the conserved HXXXD or DFGWG motifs. These *BAHD* genes were systematically renamed according to their chromosomal locations (Table S1). In summary, compared to *Arabidopsis*, the three *Brassica* species exhibited significant gene expansion in the BAHD family. Notably, the allotetraploid *B. napus* contained nearly equivalent *BAHD* numbers to the combined total of its diploid progenitors (*B. rapa* and *B. oleracea*), suggesting whole-genome duplication (WGD) events occurred during polyploidization. BAHD proteins exhibited diverse physicochemical properties, with lengths ranging from 150 to 544 amino acid residues, molecular weights varied from 16.17 to 59.65 kDa, and isoelectric points (pI) spanning 4.44 to 9.32 (detailed in Table S1).

### 2.2. Phylogenetic Analysis of BAHD Proteins in Arabidopsis and Three Brassica Species

**Figure 1:**
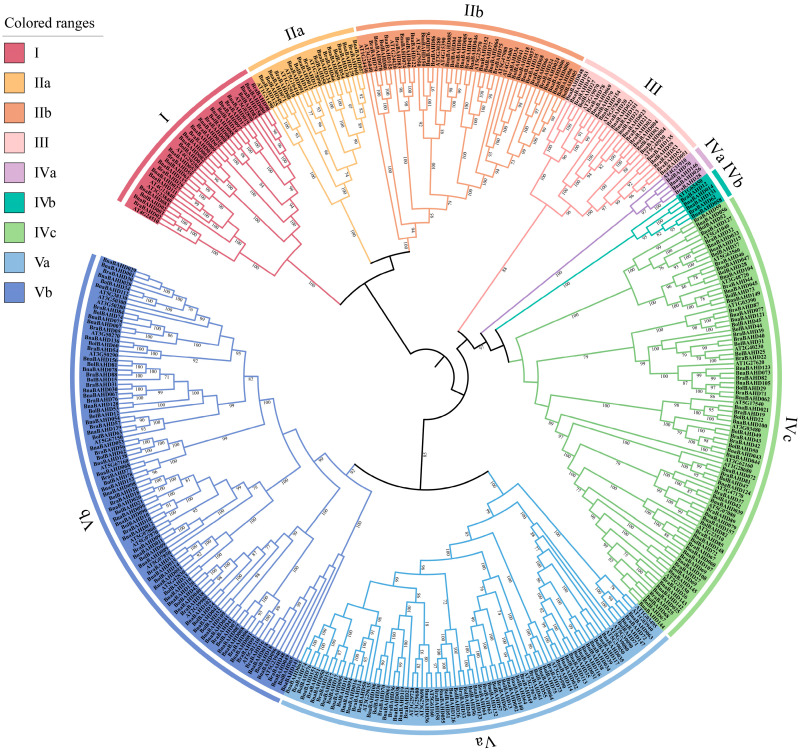
Maximum-likelihood (ML) phylogenetic tree of the BAHD proteins in *Arabidopsis* and three *Brassica* species. The sequences were categorized into five primary clades, which were further subdivided into several subclades. Each clade and subclade were distinguished by specific colors.

Cruciferous plants originated from a common ancestral species and have experienced genome duplication and fusion events during the process of evolution [30]. To better investigate the cross-species relationships of BAHD family proteins, we constructed a phylogenetic tree. It included a total of 395 *BAHD* genes (158 from *B. napus*, 83 from *B. oleracea*, 91 from *B. rapa*, and 63 from *Arabidopsis*), and was performed using the Maximum-likelihood (ML) method. Based on clustering with known *Arabidopsis* orthologs, their BAHD family genes were categorized into five distinct clades (Clades I–V; Figure 1).

Clade I contains fewer members compared to other clades (Figure 1), but 80% of its *Arabidopsis* genes, such as *AT4G24510* (*AtCER2*), *AT4G13840* (*AtCER26*), *AT3G23840* (*AtCER26-like*), and *AT5G02890*, have been well-studied. These genes are expressed in stems, leaves, flowers, and siliques, respectively, and the proteins they encode regulate the cuticular wax biosynthetic pathway by influencing very long-chain fatty acid (VLCFA) elongation [3,5,31]. Based on these findings, we speculated that *B. napus* genes in this clade (e.g., *BnaBAHD003*, *BnaBAHD027*, *BnaBAHD083* and *BnaBAHD111*) may also function in cuticular wax metabolism. Clade II, divided into subclades IIa and IIb (Figure 1), includes numerous members involved in the catalytic acetylation of alcohol molecules [19]. Notably, *AT4G15400,* within this clade, encodes the cell surface receptor kinase BRASSINOSTEROID-INSENSITIVE 1 (BRI1), which plays a critical role in maintaining brassinosteroid (BR) homeostasis and modulating photomorphogenesis, as well as other processes essential for plant growth and development [32,33]. Based on their phylogenetic placement, *BnaBAHD037* and *BnaBAHD118* may share similar functions in BR biosynthesis and signaling. Clade III, despite having the fewest members (Figure 1), contains three functionally significant *Arabidopsis* genes. *AT5G48930 (AtHCT)* is essential for monolignol biosynthesis in the lignin biosynthesis pathway, influencing plant defense and disease resistance [34,35]. *AT5G57840* plays a critical role in catalyzing the formation of glucuronosylglycerol-phenylacetic acid esters, thereby modulating phenylalanine and lipid metabolism [36]. Additionally, *AT2G19070 (AtSHT)* is crucial for *Arabidopsis* pollen development, as its encoded enzyme is specifically expressed in pollen sacs and involved in the synthesis of hydroxycinnamoyl spermidines, which are essential for pollen wall and pollen coat formation [37].

Clade IV is categorized into three subclades: IVa, IVb, and IVc (Figure 1). *AT1G65450,* in Clade IVb, is involved in *Arabidopsis* seed development by regulating double fertilization [38]. Clade IVc includes three well-characterized genes (*AT5G41040*, *AT5G63560,* and *AT3G03480*), which play key roles in lignin biosynthesis and root wax synthesis, contributing to plant defense and hydration [39,40,41]. Additionally, *AT2G25150* (*AtSDT*) and *AT2G23510* (*AtSCT*) encode spermidine conjugate synthases essential for defense and stress responses [42]. These findings suggest that orthologous genes in other species within Clade IV may also function in secondary metabolism, defense mechanisms, and stress adaptation. Clade V, the largest clade, is divided into two subclades: Va (29 *BnaBAHDs*) and Vb (41 *BnaBAHDs*) (Figure 1). Genes in this clade are closely associated with the acylation modification of plant anthocyanins and play an important role in the process of anthocyanin biosynthesis. For example, in *Arabidopsis*, *AT3G29590 (At5MAT)*, *AT1G03940 (At3AT1)*, and *AT1G03495 (At3AT2)* encode acyltransferases that catalyze the addition of acyl groups to anthocyanins, enhancing their stability and light absorption capacity. This modification consequently influences plant coloration and stress adaptability [43,44]. Given their phylogenetic location, their putative orthologs (*BnaBAHD036*, *BnaBAHD064*, and *BnaBAHD147*) may encode enzymes involved in anthocyanin acylation.

### 2.3. Analysis of Conserved Motifs and Gene Structure of the BnaBAHD Family

**Figure 2:**
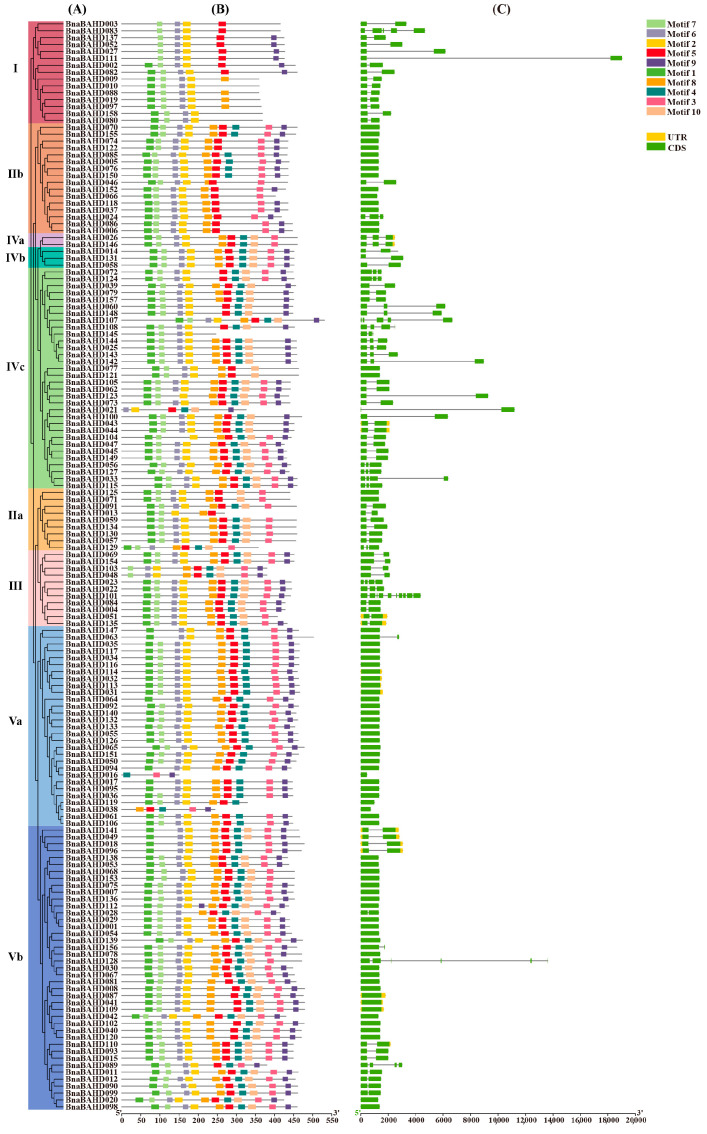
Phylogenetic trees, conserved protein motifs, and gene structure pattern in *B. napus*. (**A**) Phylogenetic analysis of BnaBAHD proteins. The coloring of each clade is consistent with that presented in Figure 1. (**B**) Conserved motifs of BnaBAHD proteins. Distinct motifs were visually represented by boxes of different colors. Specifically, motif2 corresponds to the HXXXD domain, and motif3 represents the DFGWG domain. Comprehensive sequence details for each individual motif are provided in Table S2. (**C**) Gene structure of *BAHD* gene family. Exons (coding sequences, CDS) and introns are represented by green boxes and gray lines, respectively. Yellow boxes represent upstream or downstream untranslated regions (UTR).

To comprehensively visualize the genetic relatedness within the BnaBAHD protein family and explore their evolutionary relationships, a Maximum-likelihood (ML) phylogenetic tree was constructed using the predicted amino acid sequences. Furthermore, 10 conserved motifs were identified in BnaBAHD proteins, highlighting structural features of the BAHD family members in *B. napus* (Figure 2; Table S2). Notably, every member of the BnaBAHD family contains either motif2 or motif3, with 75.3% (119 out of 158 members) harboring both of them simultaneously (Table S3). Through gene structure analysis, we determined that the sequences of motif2 and motif3 are NHAVADGTSLWMFLNSWAEIA and VASSPRFGVYGNDFGWGK, corresponding to the HXXXD and DFGWG domains, respectively.

Analysis of these conserved motifs revealed a high degree of similarity among members within the same clade. The HXXXD motif is widely present across all clades, detected in approximately 97.5% of *BnaBAHD* genes, with the exception of several genes in Clades IIa and IV (e.g., *BnaBAHD129*, *BnaBAHD016*, *BnaBAHD038*, and *BnaBAHD028*) where it is absent. In contrast, the DFGWG motif exhibits relatively lower conservation, being absent in around 22.2% of *BnaBAHD* genes. Specifically, it is entirely absent in all genes of Clade I and some genes of Clades IIa, IVc, and Va. Moreover, in certain genes, this motif is transformed into variants such as NFGWG in *BnaBAHD102*, EFGMG in *BnaBAHD110*, QFGMG in *BnaBAHD098* and *BnaBAHD099*, SFGWG in *BnaBAHD85*, DFGLG in *BnaBAHD046*, DFGFG in *BnaBAHD125*, and so on.

The analysis of the *BnaBAHD* family gene structures demonstrated that most genes in this family exhibit a similar architecture. The number of exons typically ranges from 1 to 3, accounting for 94.9% (150 out of 158) of the *BnaBAHD* family genes. Among these genes, *BnaBAHD101* is notable for having the highest number of exon (10), while *BnaBAHD128* (6), *BnaBAHD083* (5), and *BnaBAHD107* (5) also exhibit relatively complex structures (Figure 2C). Overall, within *B. napus*, most *BnaBAHD* genes within the same subclade display a high degree of structural similarity, suggesting functional conservation among subgroup members. For instance, most genes in Clades IIb and V contain only a single exon and no introns, with relatively uniform lengths typically under 2000 bp. Conversely, genes with two or more exons are predominantly clustered in other subgroups, with total lengths exceeding 2000 bp. Notably, the *BnaBAHD111* in Clade I stands out with a length approaching nearly 20,000 bp.

### 2.4. Chromosomal Localization Analysis of BnaBAHD Genes

**Figure 3:**
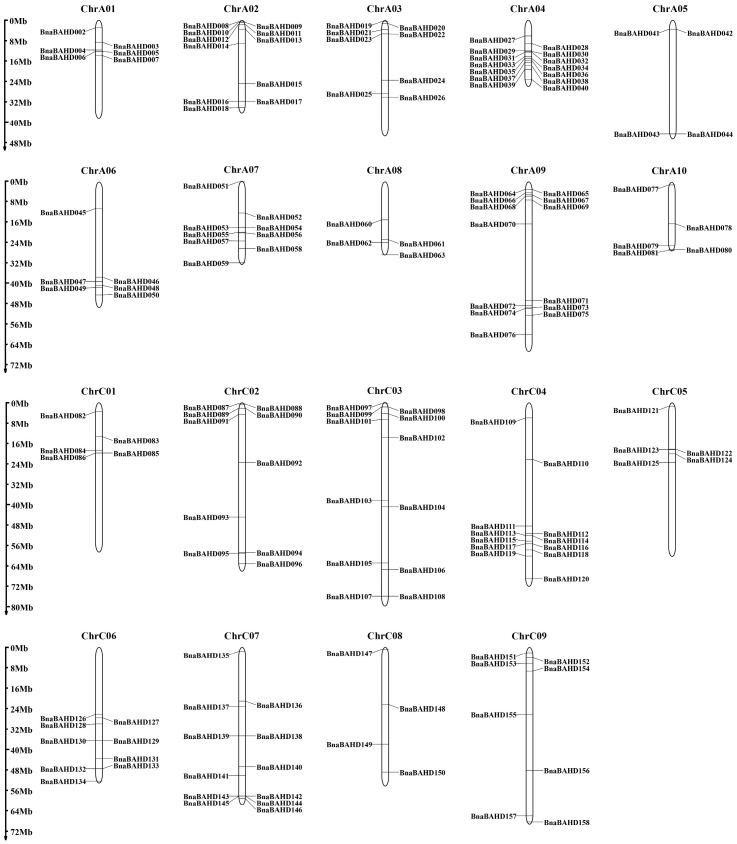
Chromosome distribution and analysis of the *BAHD* family genes in *B. napus*.

All 158 *BnaBAHD* genes were precisely mapped onto the chromosomes of *B. napus* and systematically designated as *BnaBAHD001* to *BnaBAHD158* based on their physical positions (Figure 3). Among them, *BnaBAHD001*, located on scaffold0026, was excluded due to its distinct genomic context.

Further analysis revealed that the *BnaBAHD* genes are widely distributed across the whole genome of *B. napus*. However, their distribution characteristics varied among different scaffolds, in terms of both distribution positions and densities. For example, ChrA04 and ChrA09 exhibited high gene enrichment, harboring multiple *BnaBAHD* members clustered within specific genomic intervals, with relatively short intergenic distances. This clustering pattern suggests potential functional or regulatory relationships among these genes. In contrast, ChrA05 and ChrC08 displayed sparse distribution patterns, with genes dispersed across larger chromosomal regions.

### 2.5. Identification of Gene Duplication Events Within the BAHD Gene Family of B. napus and Collinearity Analysis of BAHD Genes Among Three Brassica Species

**Figure 4:**
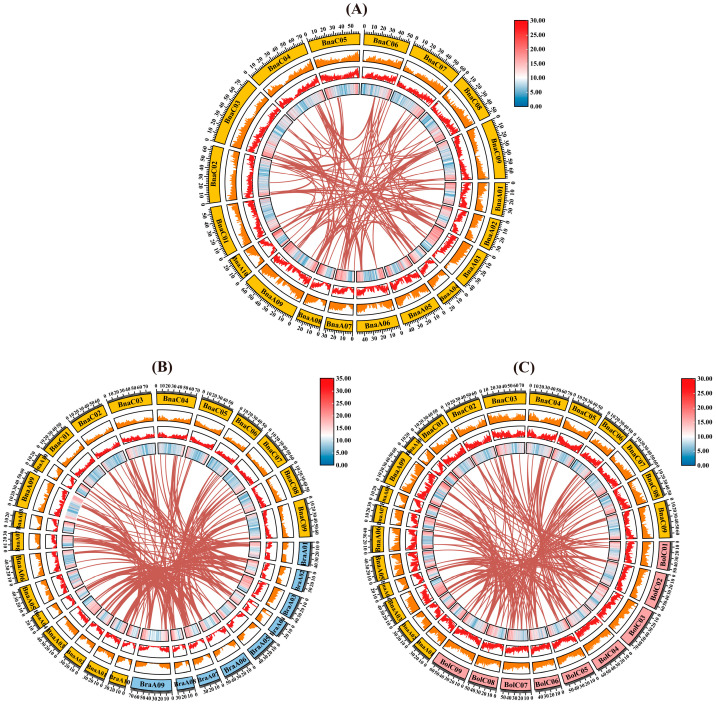
Gene duplication and collinearity analysis of *BAHD* genes in *B. napus* and its parental species. (**A**) Identification of gene duplication events within the *BAHD* family of *B. napus*. (**B**) Collinearity analysis of *BAHD* family genes between *B. napus* and *B. rapa*. (**C**) Collinearity analysis of BAHD family genes between *B. napus* and *B. oleracea*. The outermost ring displays chromosomes carrying *BAHD* genes, with distinct colors representing chromosomes from different species. The middle layer, composed of heatmaps, line plots, and bar plots, illustrates the density distribution of genes along these chromosomes. Internally, dark red lines connect homologous gene pairs, highlighting their evolutionary relationships.

To elucidate the evolutionary mechanisms underlying the expansion of the *BAHD* gene family in *B. napus*, we analyzed all 19 chromosomes (Figure 4A; Table S4). A total of 138 segmentally duplicated *BAHD* gene pairs from ancestral WGD were identified, with high-density retention regions on chromosomes ChrA02, A07, C02, C03, C04, and C09.

To further trace the evolutionary origins, we investigated collinear relationships between *B. napus* and its diploid progenitors, *B. rapa* and *B. oleracea* (Figure 4B,C; Tables S5 and S6). Comparative synteny analysis revealed that 133 *BAHD* genes of *B. napus* exhibited collinearity with 63 orthologs in *B. oleracea* and 75 orthologs in *B. rapa*, forming 196 and 218 orthologous pairs, respectively. Nearly 50% of the parental genes in *B. rapa* were targeted to BnaA02, A04, A07, A09, C03, and C07 chromosomes, whereas more than 50% of the parental genes in *B. oleracea* mapped to *B. napus* chromosomes BnaA02, A04, A09, C02, C03, C04, and C09 (Figure 4B,C). These findings demonstrate that homologous *BAHD* gene pairs are widely distributed across the genomes of the allotetraploid *B. napus* and its diploid ancestors, *B. rapa* and *B. oleracea*, underscoring the conservation and diversification of the BAHD gene family during evolution.

### 2.6. Selective Pressure Analysis of BAHD Genes in B. napus, B. oleracea, and B. rapa based on Ka/Ks Ratio

During the long-term evolution of genes, nucleotide variations can lead to two distinct outcomes depending on whether they alter the amino acid sequence. Nonsynonymous substitutions, which modify the amino acid sequence, can significantly impact protein structure and function [45]. In contrast, synonymous substitutions, which do not change the amino acid sequence, generally have minimal effects on protein function [46].

The Ka/Ks ratio, a critical parameter for assessing selective pressure in gene evolution, refers to the ratio of the nonsynonymous substitution rate (Ka) to the synonymous substitution rate (Ks) [47]. By calculating the Ka/Ks ratios of each gene pair, we found that most values ranged from 0.1 to 0.4, with only a small fraction exceeding 1 (Tables S4–S6). Specifically, in the collinear gene pairs between *B. napus* and *B. oleracea*, approximately 3.52% of the Ka/Ks ratios were greater than 1. Similarly, in the collinear pairs between *B. napus* and *B. rapa*, about 1.38% of the ratios exceeded 1. These values above 1 indicate positive selection, suggesting that amino acid changes in these genes confer evolutionary advantages and are favored by natural selection.

Conversely, for all segmental duplications within *B. napus* and the majority of collinear gene pairs among *B. napus*, *B. oleracea*, and *B. rapa*, the Ka/Ks ratios were less than 1 (Tables S4–S6). This observation demonstrates that these homologous gene pairs have predominantly undergone strong purifying selection during the evolution of the three *Brassica* species. Purifying selection acts to eliminate harmful amino acid changes, thereby maintaining the stable function of genes over evolutionary time.

### 2.7. Cis-Acting Element Analysis of BnaBAHD Promoters

**Figure 5:**
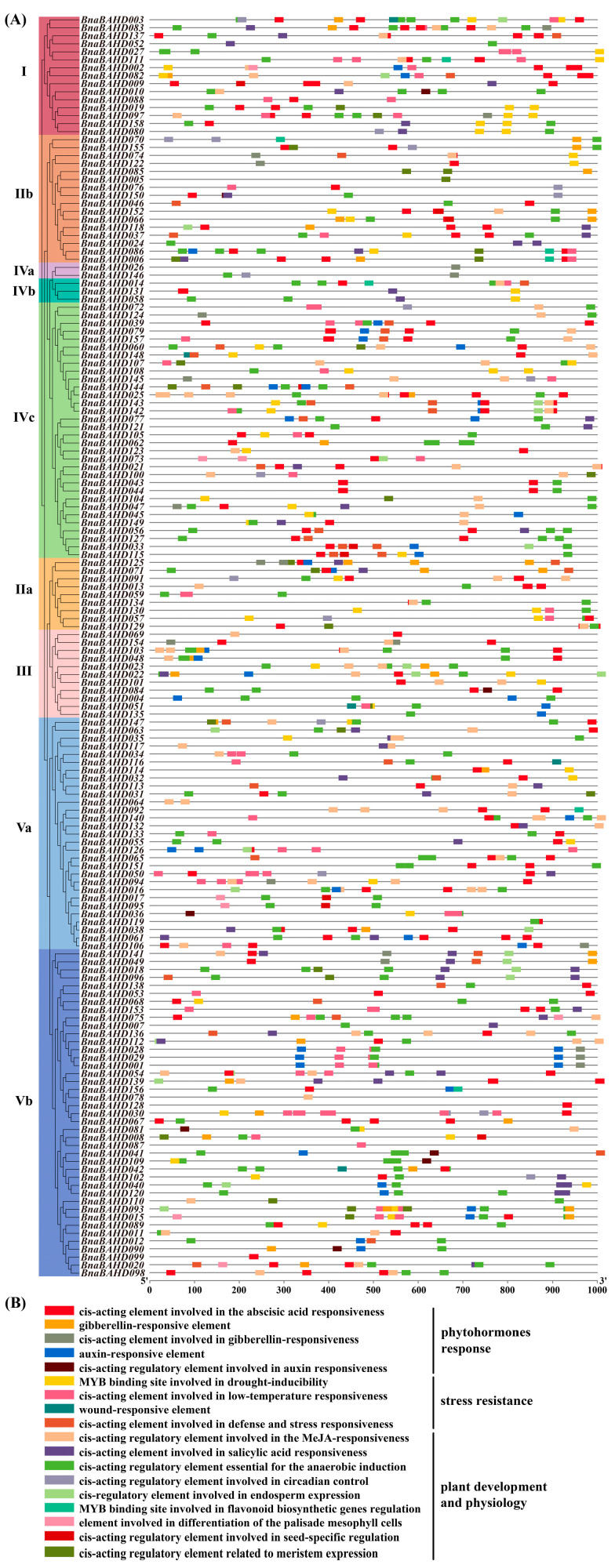
Cis-acting elements in the promoters of *BnaBAHDs.* (**A**) Spatial distribution of cis-elements (marked with different colored boxes) within the promoters. (**B**) Functional classification of cis-elements, with categories annotated on the right.

A comprehensive analysis of cis-acting elements was performed within the 1000 bp upstream regions of transcription start sites across 158 *BnaBAHD* genes in *B. napus* (Figure 5A). These promoters were characterized by three major functional categories of cis-elements: phytohormone responsiveness, abiotic stress adaptation, and growth regulation (Figure 5B). Notably, abundant hormone-responsive elements associated with abscisic acid (ABA), gibberellin (GA), and auxin suggest potential roles of *BnaBAHDs* in phytohormone-mediated signaling pathways. Additionally, the identification of four distinct abiotic stress-responsive cis-element types, along with elements linked to salicylic acid (SA) responsiveness, flavonoid biosynthetic regulation, and methyl jasmonate (MeJA) responsiveness, implies critical functions of these genes in plant defense mechanisms and adaptation to biotic/abiotic stresses [20,48,49,50]. Collectively, our findings demonstrate the pleiotropic roles of *BnaBAHD* genes in coordinating developmental and stress-adaptive processes in *B. napus*, revealing their mechanistic contributions to phytohormone signaling and stress resilience.

### 2.8. Expression Profiles of BAHD Genes in B. napus Under Nitrogen and Phytohormone Treatment

**Figure 6:**
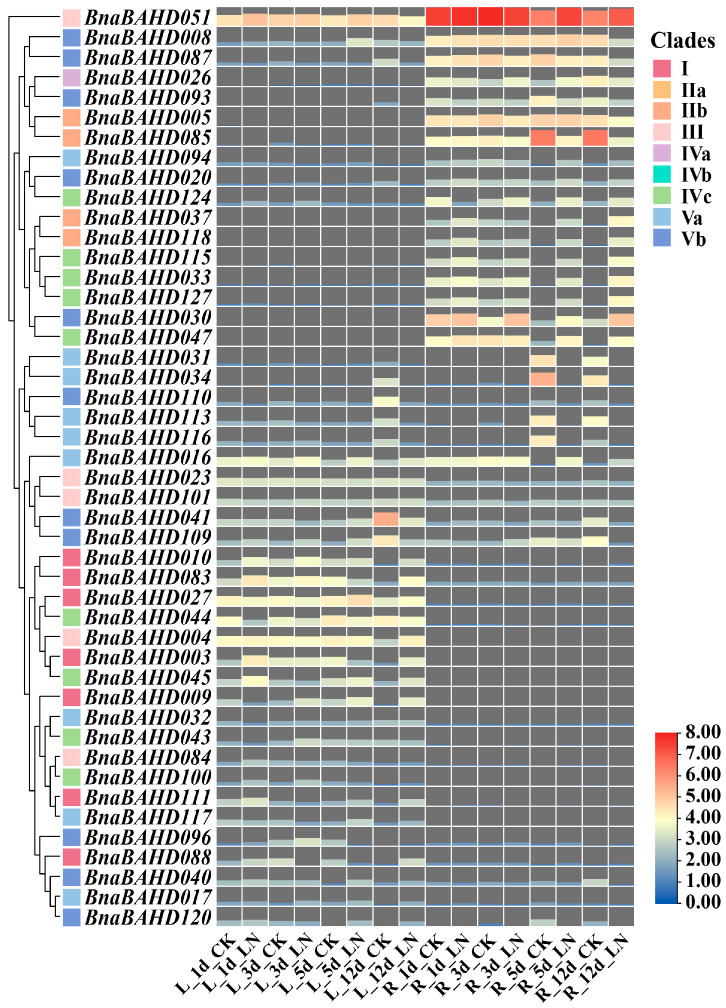
Expression profiles of *BnaBAHDs* under nitrogen treatment. The expression profiles of each *BnaBAHD* were normalized using Log_2_ transformation (FPKM + 1). Color intensity and area size of tiles correspond to expression levels. L, leaves; R, roots; CK (control), normal nutrient solution with no nitrogen deficiency; LN, low-nitrogen treatment. FPKM, Fragments Per Kilobase of exon model per Million mapped fragments.

**Figure 7:**
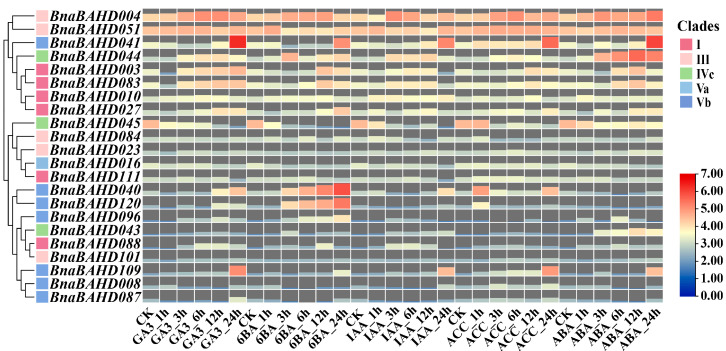
Expression profiles of *BnaBAHDs* under phytohormone treatments. The expression profiles of each *BnaBAHD* were normalized using Log_2_ transformation (FPKM + 1). Color intensity and area size of tiles correspond to expression levels. CK (control), normal nutrient solution with no phytohormone treatments. GA3, gibberellins; 6BA, 6-benzyladenine; IAA, indole-3-acetic acid; ACC, 1-aminocyclopropanecarboxylic acid; ABA, abscisic acid. Time labels (1 h, 3 h, 6 h, 12 h, 24 h) indicate hours post-treatment.

With the published RNA-Seq data from the BnaGADB database, we systematically analyzed the expression patterns of *BAHD* genes in *B. napus* under abiotic stresses, including nitrogen limitation and phytohormone treatments (GA3: gibberellins; 6-BA: 6-benzyladenine; IAA: indole-3-acetic acid; ACC: 1-aminocyclopropanecarboxylic acid; ABA: abscisic acid) (Figure 6 and Figure 7).

The majority of *BnaBAHDs* exhibited significant upregulation in roots or leaves under low-nitrogen (LN) conditions, with clade-specific expression patterns (Figure 6). For example, *BnaBAHD124/115/033/127/047* (Clade IVc), *BnaBAHD037/118* (Clade IIb), and *BnaBAHD094/020/030* (Clade V) showed pronounced induction in leaves under low-nitrogen (LN) stress, particularly at 5/12 days post-treatment. Notably, nearly all Clade I members and partial Clade V genes (e.g., *BnaBAHD040/120*) displayed elevated expression levels in roots under LN compared to controls (CK). These observations suggest that the upregulation of *BnaBAHDs* under nitrogen-deficient conditions may enhance the nitrogen use efficiency and stabilize metabolic homeostasis of *B. napus* under stress, thereby improving its adaptability to nitrogen limitation. Distinct tissue-specific expression patterns were observed among *BnaBAHDs* in *B. napus*: certain genes (e.g., *BnaBAHD051*) displayed constitutively high expression in both roots and leaves under both control and low-nitrogen conditions, whereas the majority exhibited strict organ-specific expression, being predominantly localized to either roots or leaves.

To elucidate the regulatory roles of *BnaBAHDs* under phytohormone induction, we analyzed their expression dynamics in root tissues of five-leaf stage Zhongshuang11 (ZS11, *Brassica napus*) under exogenous phytohormone treatments (Figure 7). The results demonstrated that Clade Vb members (BnaBAHD041/190) exhibited highly significant upregulation upon 24 h exposure to all five phytohormones (GA_3_, 6-BA, IAA, ACC, ABA). Notably, *BnaBAHD040/120,* within Clade Vb, exhibited pronounced hypersensitivity to 6-BA, with transcript levels progressively increasing throughout the 1–24 h treatment duration and peaking at 24 h. In contrast, partial Clade I members (e.g., *BnaBAHD003/083*) showed unimodal expression patterns under 6-BA, IAA, ACC, and ABA treatments: their expression initially increased between 3 and 12 h, peaked at 12 h, and subsequently declined by 24 h. Among the highly expressed *BnaBAHDs* in Figure 7, Clade I/IVc/Vb genes displayed marked hormone-responsive expression dynamics, whereas Clade III members exhibited relatively minimal transcriptional changes across treatments. Collectively, *BnaBAHDs* in Clade Vb exhibited the strongest sensitivity to these phytohormones, suggesting their potential role in enhancing abiotic stress adaptation through nutrient and metabolic regulation.

### 2.9. Analysis of Expression Patterns of BAHD Family in B. napus

**Figure 8:**
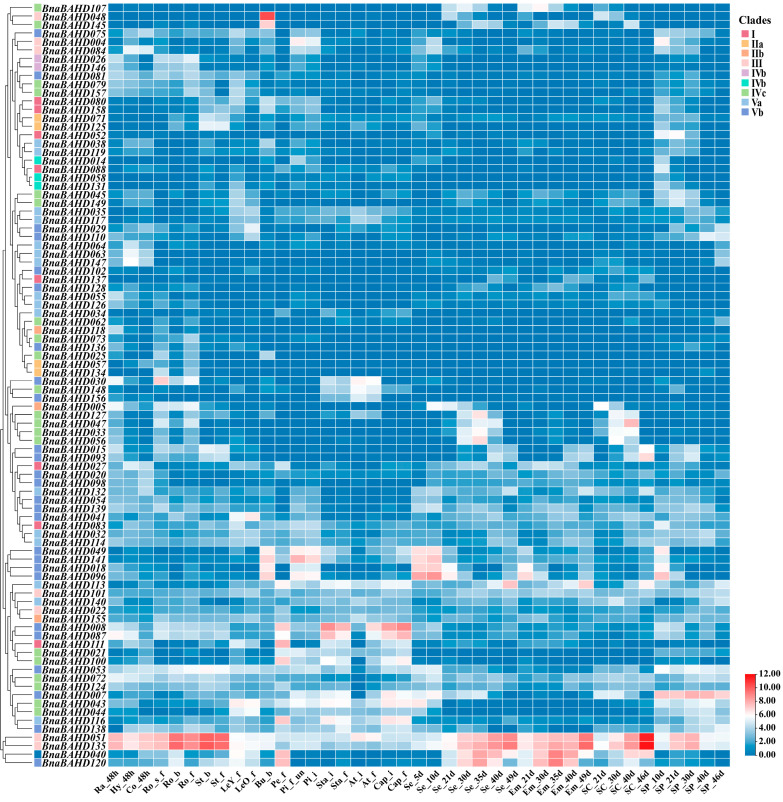
Expression profiles of *BnaBAHDs* across different tissues and organs. The expression profiles of each *BnaBAHD* were normalized using Log_2_ transformation (FPKM + 1). Ra, radicle; Hy, hypocotyl; Co, cotyledon; Ro, root; St, stem; LeY, Leaf Young; LeO, Leaf Old; Bu, bud; Pe, petal; Pi, pistil; Sta, stamen; At, anther; Cap, capillament; Se, seed; Em, embryo; SC, seed coat; SP, silique pericarp; 48 h, 48 h after seed germination; s_f, seedling stage under field cultivation condition; b, bud stage; f, full-bloom stage; un, unpollinated; i, initial flowering stage.

To investigate the potential biological functions of *BnaBAHD* genes in the growth and development of *B. napus*, we analyzed the spatiotemporal expression patterns of these annotated genes based on the transcriptome data from multiple tissues and organs of ZS11 cultivar. The comprehensive expression profiling revealed that these genes do not only extensively participate in biological regulation across various developmental stages of *B. napus* but also exhibit functional roles in diverse tissues and organs, including radicle, hypocotyl, cotyledon, root, stem, leaf, bud, petal, pistil, stamen, anther, filament, seed, embryo, seed coat, and silique pericarp (Figure 8; Table S7). With the exception of *BnaBAHD066* and *BnaBAHD130* in Clade II, which showed no detectable expression in all examined tissues, the remaining *BnaBAHDs* exhibited distinct tissue specificity.

Hierarchical clustering analysis further demonstrated that *BnaBAHD* members within the same clade exhibited conserved expression patterns. For instance, orthologous genes in Clade III (*BnaBAHD051* and *BnaBAHD135*) were broadly expressed across nearly all tissues, with peak accumulation in root, stem, seed, embryo, and seed coat. Clade IV members (*BnaBAHD047*, *BnaBAHD127*, *BnaBAHD033*, and *BnaBAHD056*) were predominantly activated during mid-to-late developmental phases of seed and seed coat. Within Clade V, orthologs *BnaBAHD040* and *BnaBAHD120* displayed marked expression in mid-to-late seed/embryo development and petals; *BnaBAHD008* and *BnaBAHD087* were enriched in stamen, filament, and petals; while *BnaBAHD049*/*BnaBAHD141*/*BnaBAHD018*/*BnaBAHD096* preferentially expressed in buds, pistils, and early seed development. In contrast, Clade I and Clade II genes generally showed low expression levels across all examined tissues.

Collectively, these findings suggest critical roles of BAHD acyltransferase family genes in regulating developmental processes in *B. napus*, particularly in seed maturation, floral organ differentiation, and tissue specialization.

### 2.10. Functional Analysis of BnaBAHD040 and BnaBAHD120 via Transient Expression in Nicotiana benthamiana

Given the agricultural importance of seeds in *B. napus*, our study prioritized two candidate genes (*BnaBAHD040* and *BnaBAHD120*) highly specifically expressed in seeds and embryos for functional validation, based on the transcriptomic sequencing data in Section 2.9. To clarify the catalytic functions of these two BAHD acyltransferase family members, we conducted transient expression experiments in *N. benthamiana*.

**Figure 9:**
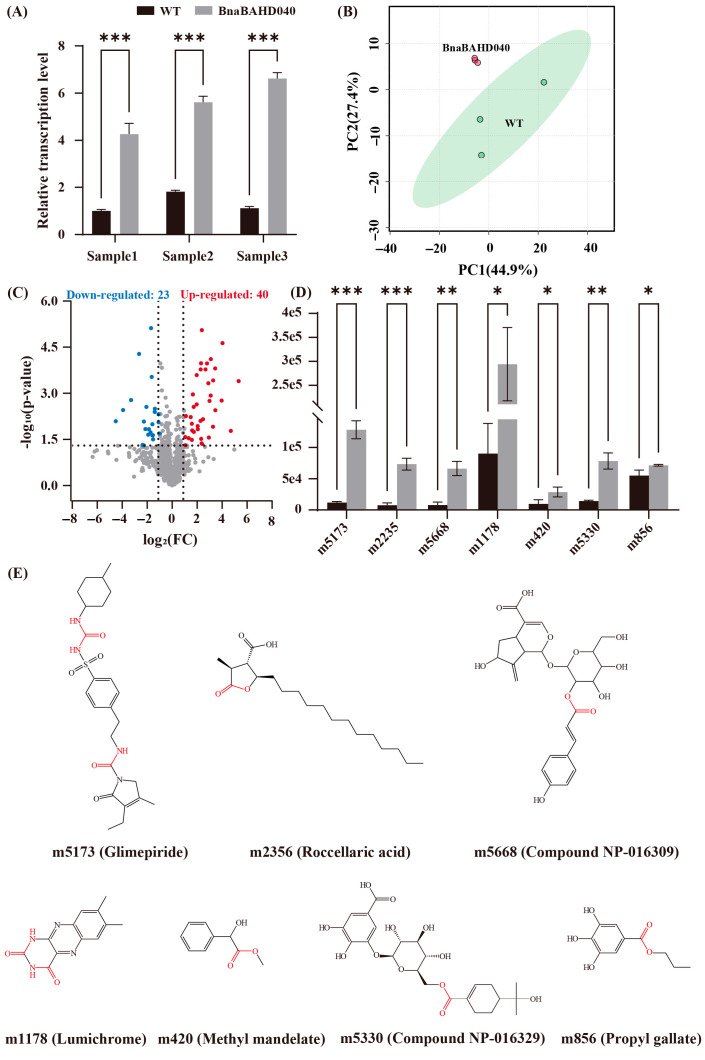
Functional characterization of *BnaBAHD040* through transient expression in *N. benthamiana*. (**A**) qRT-PCR analysis of *BnaBAHD040* expression levels. (**B**) Principal component analysis (PCA) of metabolome data from *N. benthamiana* leaves. The first two principal components accounted for 72.3% of total variance (PC1: 44.9%, PC2: 27.4%). (**C**) Volcano plot analysis of differentially accumulated metabolites. The red points represent upregulated metabolites and the blue points represent downregulated metabolites. Dashed lines indicate significance thresholds. (**D**) Relative abundance of seven significantly upregulated acylated metabolites. *, *p* < 0.05; **, *p* < 0.01; ***, *p* < 0.001. (**E**) Chemical structures of seven significantly upregulated acylated metabolites. Ester (-COO-) and amide (-CONH-) bonds, the characteristic features of acylated metabolites, are highlighted in red.

qRT-PCR analysis revealed that the expression levels of *BnaBAHD040* and *BnaBAHD120* in *N. benthamiana* leaves peaked in Sample3 and Sample1, respectively, with maximum increases of approximately 6-fold and 13-fold compared to controls, indicating their successful expression (Figure 9A, Supplementary Figure S1A). Then, we selected the tobacco samples with the highest expression levels for UPLC-HESI-MS/MS metabolomics analysis. Principal component analysis (PCA) demonstrated significant metabolic separation between transgenic and control samples (Figure 9B, Supplementary Figure S1B). Differential metabolite analysis indicated that *BnaBAHD040* overexpression significantly upregulated 40 metabolites and downregulated 23 metabolites, while *BnaBAHD120* overexpression induced significant upregulation of 15 metabolites and downregulation of 79 metabolites (Figure 9C, Supplementary Figure S1C).

Subsequently, public databases were employed to annotate all metabolites, with structural elucidation focusing on the upregulated metabolites. The identification results showed that *BnaBAHD040* enhanced the accumulation of seven acylated derivatives, including five *O*-acylated products characterized by ester bonds and two *N*-acylated compounds bearing amide bonds (Figure 9D,E; Table S9). Similarly, *BnaBAHD120* overexpression promoted the accumulation of six acylated metabolites, all exhibiting canonical *O*-acylation signatures except for m1178 (Supplementary Figure S1D,E; Table S9).

## 3. Discussion

The BAHD acyltransferase family constitutes one of the most expansive enzyme superfamilies in land plant genomes, functioning as versatile catalysts that mediate acyl transfer reactions essential for synthesizing structurally diverse secondary metabolites [51]. Members of this family play pivotal roles in plant growth, reproduction, and defense mechanisms, including but not limited to the biosynthesis of aromatic volatile esters, alkaloid modification, and production of phenolic amides linked to disease and insect resistance [16,25,52,53]. Evolutionary genomic analyses reveal a striking expansion pattern within this gene family. While basal algae genomes contain only 1–5 copies, substantial gene duplication events have occurred during terrestrial plant evolution, with gymnosperms and angiosperms typically harboring 50–200 members [54]. For example, 123 *TwBAHDs* and 112 *CsBAHDs* were identified in *Taxus wallichiana* and *Camellia sinensis*, respectively [15,52]. This suggests that BAHD family members have accumulated extensive tandem duplications during plant evolution, functionally enabling metabolic diversification and enhanced environmental adaptability.

Through a comprehensive genome-wide analysis, 158 *BnaBAHD* genes were identified in the allotetraploid *B. napus*, a count closely approaching the combined homologs of its diploid progenitors *B. rapa* (91 *BraBAHDs*) and *B. oleracea* (83 *BolBAHDs*). This near-additive retention pattern suggests that whole-genome duplication (WGD) events shaped the BAHD family during *B. napus* allopolyploidization. Notably, the slightly lower gene count in *B. napus* compared to the ancestral sum, combined with Ka/Ks evolutionary selection pressure analysis, indicates significant purifying selection acting on the BAHD family during polyploidization. This selection likely eliminated deleterious mutations and promoted the loss of redundant genes, thereby maintaining genomic stability in *B. napus* [55].

Based on previous phylogenetic classifications of BAHD acyltransferases in angiosperms, we classified the identified *BAHDs* in *Arabidopsis* and three *Brassica* species into five clades and constructed a phylogenetic tree (Figure 1) [14,17,56]. Functional annotation of characterized *Arabidopsis* orthologs revealed several possible clade-specific roles: Clade I mediates cuticular wax biosynthesis; Clade II regulates brassinosteroid signaling and alcohol acetylation; Clade III participates in lignin biosynthesis, phenylpropanoid/lipid metabolism, and pollen development; Clade IV contributes to seed maturation and stress-adaptive compound synthesis; and Clade V acts in plant anthocyanin acylation to modulate pigmentation and stress resilience. Comparative genomic analysis revealed significant intron reduction in *BnaBAHDs* compared to algal *BAHDs*, which retain multiple introns within their single catalytic domains [51]. Notably, approximately 80% of *BnaBAHDs* retain only one intron or are entirely intronless (Figure 2C), aligning with the evolutionary trajectory of intron loss during plant terrestrialization [51].

Building on the phylogenetic and genomic insights, our findings demonstrate that *BAHD* genes in *B. napus* are enriched with cis-regulatory elements linked to phytohormone signaling (ABA, GA, auxin) and abiotic stress adaptation, suggesting their potential roles in mediating stress-responsive pathways and hormone crosstalk. RNA-Seq analyses validated these predictions, revealing clade-specific expression dynamics under nitrogen limitation, with several pronounced upregulations of *BnaBAHDs* in roots or leaves during prolonged low-nitrogen stress (Figure 6). Hormone-responsive expression patterns further highlighted functional divergence, especially the hypersensitivity of Clade Vb members (e.g., *BnaBAHD040/120*) to 6-BA and their sustained induction under multiple phytohormones (Figure 7). These results suggest that *BnaBAHDs*, particularly Clade Vb, likely contribute to phytohormone signal integration and nitrogen stress resilience through coordinated transcriptional regulation in *B. napus*.

To further explore the functional implications of these regulatory and expression features, we selected two homologous seed-specific Clade V BAHD acyltransferase genes, *BnaBAHD040* (located on chromosome BnaA04 of the A_n_ subgenome derived from *B. rapa*) and *BnaBAHD120* (located on chromosome BnaC04 of the C_n_ subgenome derived from *B. oleracea*), in *B. napus,* for detailed analysis. Their functional roles and divergence in acylated metabolite regulation were investigated through transient overexpression in *N. benthamiana* combined with UPLC-HESI-MS/MS metabolomic analysis. This approach revealed that *BnaBAHD040* significantly increased the content of seven acylated metabolites (*p* < 0.05), while *BnaBAHD120* only caused a non-significant increase in six metabolites (Figure 9, Figure S1). We suppose that the functional divergence between these paralogs may arise from their distinct spatiotemporal expression patterns across tissues. Notably, *BnaBAHD040* displayed dramatically higher expression levels during late seed developmental stages compared to *BnaBAHD120* (2.2-fold higher at 40 days after flowering and 11.9-fold higher at 49 days) (Table S7). Furthermore, our findings provide novel evidence supporting the A_n_ subgenome (derived from *B. rapa*) in exhibiting higher gene expression levels compared to the C_n_ subgenome (derived from *B. oleracea*) in *B. napus* [57].

Previous studies have demonstrated that BAHD acyltransferases catalyze the formation of *O*-acylated (ester bond-containing) and *N*-acylated (amide bond-containing) metabolites [12]. Through metabolomic profiling and structural characterization, we observed that the majority of acylated metabolites from BnaBAHD040/120 were *O*-acylated derivatives, with a smaller proportion identified as *N*-acylated compounds, all exhibiting characteristic features of typical BAHD-catalyzed products (Figure 9 and Supplementary Figure S1). Notably, the synthesis of m856 (propyl gallate) likely employs galloyl-CoA, a canonical acyl donor proposed in prior studies, as the acyl donor and n-propanol as the acyl acceptor [58]. Furthermore, this enzyme group exhibits significant substrate promiscuity, typically generating a broad array of structurally diverse acylated metabolites that participate in multiple biochemical pathways [51,59]. Several of these metabolites have been functionally linked to plant defense mechanisms: Propyl gallate (m856) enhances plant disease resistance by inhibiting pathogen biofilm formation and activating antioxidant systems; Lumichrome (m1178) improves stress tolerance through modulation of rhizosphere interactions; and Ethyl chrysanthemate (m685) participates in insect repellency and insecticidal activity [60,61,62,63,64]. Therefore, we hypothesize that these metabolites may enhance the environmental adaptability of *B. napus* by playing roles in responding to both biotic and abiotic stresses.

This study uncovered the heterologous expression of specific BAHD acyltransferase family members in *B. napus*, explicitly demonstrating their catalytic roles in the biosynthesis of acylated metabolites. These findings provide direct experimental evidence elucidating the functional contributions of *BnaBAHDs* to plant secondary metabolism. Furthermore, this research establishes a critical foundation for leveraging BAHD family genes in rapeseed improvement. Through genetic engineering, these enzymes can be strategically utilized to develop novel *B. napus* cultivars with enhanced stress resilience and improved yield.

## 4. Materials and Methods

### 4.1. Source of Plants and Data

The BAHD protein sequences of *A. thaliana*, *B. rapa* (Bra, genotype AA), *B. oleracea* (Bol, genotype CC), and *B. napus* (Bna, genotype AACC) were downloaded from the TAIR database (https://www.arabidopsis.org/ (accessed on 6 May 2024)), the Brassicaceae Database (BRAD, http://brassicadb.cn/ (accessed on 6 May 2024)), and the multi-omics database for *Brassica napus* (BnIR, https://yanglab.hzau.edu.cn/ (accessed on 6 May 2024)) [65,66].

### 4.2. Identification and Annotation of BAHD Family Gene Sequences

First, using the BAHD family characteristic domain (Pfam: PF02458) as a query template, a total of 63 candidate genes belonging to the BAHD family were retrieved and identified from the *Arabidopsis* protein sequences through the Hidden Markov Model (HMM) search method, with the E-value threshold strictly less than 1 × 10^−10^ [67]. For *Brassica* species (*B. rapa*, *B. oleracea,* and *B. napus*), we similarly first performed an HMM search on their respective proteomes using TBtools’ HMM (v2.136), followed by bidirectional BLASTp (http://ftp.ncbi.nlm.nih.gov/blast/executables/blast+/LATEST/ (accessed on 27 June 2024)) alignments between the protein sequences of *Arabidopsis* and each of the three *Brassica* species to identify homologous genes [68]. Notably, only sequences demonstrating high homology in both the forward and reverse alignments were retained, and then candidates from both approaches were combined. Domain analyses were carried out using Pfam (http://pfam-legacy.xfam.org/ (accessed on 3 July 2024)) and NCBI CD-search (https://www.ncbi.nlm.nih.gov/Structure/cdd/wrpsb.cgi (accessed on 3 July 2024)), and screening was conducted using the characteristic domain PF02458 as the standard [67,69]. In addition, the online program Multiple Expectation Maximization for Motif Elucidation (MEME, http://meme-suite.org/tools/meme (accessed on 15 January 2025)) was utilized to detect the characteristic conserved motifs (HXXXD and DFGWG), and genes with incomplete structures were discarded [70]. Ultimately, we identified 91 *BraBAHDs*, 83 *BolBAHDs,* and 158 *BnaBAHDs* possessing a confirmed PF02458 domain, at least one conserved motif (HXXXD or DFGWG), and full-length structure. The online software tools TBtools-II (Toolbox for Biologists v2.136) and Expasy (https://www.expasy.org/ (accessed on 27 January 2025)) were utilized to predict fundamental properties of each BAHD protein sequence, including its chromosomal location, length (number of amino acid residues), molecular weight (MW), and isoelectric point (pI) [71].

### 4.3. Phylogenic Analysis of the BAHD Family Members

We used TBtools’ One Step Build an ML Tree to construct two Maximum-likelihood (ML) phylogenetic trees. One tree included genes from the BAHD family of four species: *A.thaliana*, *B. rapa*, *B. oleracea,* and *B. napus*, to analyze their overall phylogenetic relationships. The other focused on the *BAHD* genes in *B. napus* for a detailed study of these genes’ evolution. Then, we utilized the iTol online site (https://itol.embl.de/ (accessed on 14 March 2025)) for visualization and beautification [72]. To better visualize evolutionary relationships, we also rooted the phylogenetic trees at the midpoint of all branches.

### 4.4. Conserved Motif Identification and Gene Structure Analysis of BnaBAHDs

The conserved motifs of BnaBAHD proteins were predicted using the online program MEME (http://meme-suite.org/tools/meme (accessed on 15 January 2025)) [70]. The parameters were set as follows: the maximum number of motifs was 10, the motif length was restricted between 6 and 300 amino acid residues, and all other parameters were kept as default. The gene structure (exon–intron structure) and motif patterns were visualized in conjunction with the phylogenetic tree using the Gene Structure Display function in TBtools.

### 4.5. Chromosomal Localization and Colinearity Analysis of BnaBAHD Genes

The chromosomal lengths and genomic coordinates (including chromosome numbers, start positions, and end positions) of the *BnaBAHD* genes were extracted from the *B. napus* genome sequence data available in the BnIR database (https://yanglab.hzau.edu.cn/ (accessed on 6 May 2024)). These genes were then mapped to their corresponding chromosomes using the MapGene2Chrom (MG2C) online tool (http://mg2c.iask.in/mg2c_v2.1/ (accessed on 1 February 2025)) [66,73]. To elucidate the duplication patterns of the *BnaBAHD* genes and their collinear relationships with *BraBAHD* and *BolBAHD* genes, TBtools’ One Step MCScanX (v2.136) tool was employed [74], and the results were visualized using TBtools’ Advanced Circos (v2.136) [75]. Furthermore, to assess the selective pressures acting on these genes during evolution, the nonsynonymous substitution rate (Ka), synonymous substitution rate (Ks), and Ka/Ks ratio were calculated using the TBtools Simple Ka/Ks Calculator.

### 4.6. Cis-Element Analysis of BnaBAHD Promoters

To investigate the cis-regulatory elements of *BnaBAHDs*, the upstream 1000 bp sequences of these genes were extracted using TBtools’ Gtf/GFF3 Sequences Extract. These promoter regions were subsequently analyzed in the PlantCARE database (http://bioinformatics.psb.ugent.be/webtools/plantcare/html/ (accessed on 19 April 2025)) for cis-element identification [76]. Predicted elements were graphically represented through TBtools’ Simple BioSequence Viewer.

### 4.7. Analysis of Gene Expression Profile of BnaBAHD Family Genes

To elucidate BAHD gene functions in abiotic stress adaptation, hormonal responses, and tissue-specific regulation, we analyzed RNA-Seq datasets from two independent sources: (1) the BnaGADB database (*Brassica napus*. L Genome Annotation Database, http://www.bnagadb.cn/ (accessed on 15 April 2025)), providing root transcriptomes under nitrogen limitation and phytohormone treatments (GA3, 6-BA, IAA, ACC, ABA), and (2) BrassicaEDB (A Gene Expression Database for *Brassica* Crops, https://brassica.biodb.org/ (accessed on 28 July 2024)) [77], containing multi-organ expression profiles (roots, stems, leaves, flowers, siliques) of elite inbred line ZS11. All datasets were normalized to log2-transformed (FPKM value +1) to enhance visualization and comparability of *BnaBAHDs* expression profiles across different treatments and tissues.

### 4.8. Gene Cloning and Overexpression Vector Construction

Genomic DNA (gDNA) was extracted from young leaves of *B. napus* cultivar Zhongyou 821 (ZY821, *B. napus*) using the Dzup (Plant) Genomic DNA Isolation Reagent (Sangon Biotech, Shanghai, China). Total RNA was subsequently reverse-transcribed into complementary DNA (cDNA) with the HiScript IV All-in-One Ultra RT SuperMix for qPCR (Vazyme Biotech, Nanjing, China). Subsequently, the coding sequences of BnaBAHD040 and BnaBAHD120 were amplified from ZY821 cDNA by polymerase chain reaction (PCR) using gene-specific primers. The pNC-Cam33FC overexpression vector was constructed using the Nimble Cloning Kit (NC Biotech, Hainan, China). Related sequences referenced the ZY821 genome reported in our laboratory’s previous study [78].

### 4.9. Transient Transformation in N. benthamiana and qRT-PCR Expression Analysis

*Agrobacterium tumefaciens* GV3101 (pMP90) strains harboring recombinant vectors (pNC-Cam33FC-BnaBAHD040/120) or empty vector (control) were infiltrated into the left (control) and right (recombinant) sides of fully expanded *N. benthamiana* leaves using a needleless syringe. After 24 h dark incubation followed by 24 h light exposure, infiltrated leaf tissues were collected for total RNA extraction (EZ-10 DNAaway RNA Mini-Preps Kit, Sangon Biotech). Total RNA was reverse-transcribed to cDNA (HiScript IV All-in-One RT SuperMix, Vazyme) and subjected to qRT-PCR analysis on a Bio-Rad CFX96 system with *Nb26S* as the internal reference [79]. Relative expression levels of *BnaBAHD040* and *BnaBAHD120* were quantified using the 2^−ΔΔCt^ method, with three technical replicates per sample [80]. Statistical significance was assessed by two-way ANOVA (ns, not significant; *, *p* < 0.05; **, *p* < 0.01; ***, *p* < 0.001). Primer sequences are listed in Table S8.

### 4.10. Metabolomic Profiling and Structural Characterization of Acylated Metabolites

Raw metabolites were extracted from *N. benthamiana* leaf tissues exhibiting peak *BnaBAHD040* or *BnaBAHD120* expression (as determined by qRT-PCR in Section 4.8), using the previously described method [81,82,83]. Extracts were analyzed via ultrahigh-performance liquid chromatography-heated electrospray ionization-tandem mass spectrometry (UPLC-HESI-MS/MS) with three biological replicates per sample [84]. Subsequently, raw data were processed MS-DIAL v4.6 with three databases—MoNA, MSMS_Public_EXP_VS17, and MSMS_Public_ExpBioInsilico_VS17 (https://systemsomicslab.github.io/compms/msdial/main.html#MSP (accessed on 17 January 2025)) [85]. The parameters of MS-DIAL were adjusted following the previously published studies [84].

Principal component analysis (PCA) and volcano plots were generated using GraphPad Prism 10.1.2 to visualize metabolic profile divergence and identify differentially accumulated metabolites (thresholds: |log_2_(fold change)| > 1, *p* < 0.05). Relative abundances of seven significantly upregulated acylated metabolites were plotted as bar charts (mean ± SD, *n* = 3). Chemical structures were retrieved from PubChem (https://pubchem.ncbi.nlm.nih.gov/ (accessed on 5 February 2025)) in SMILES format and drawn using KingDraw 3.0 (www.kingdraw.cn (accessed on 22 January 2025)), with ester (-COO-) and amide (-CONH-) bonds highlighted in red.

## 5. Conclusions

This study systematically elucidates the evolutionary dynamics and functional diversification of the BAHD acyltransferase family in *B. napus*. Genome-wide analysis identified 158 *BnaBAHD* genes, classified into five phylogenetically distinct subclades, with their expansion primarily driven by whole-genome duplication (WGD) and purifying selection during allopolyploidization. Cis-acting element analysis revealed significant enrichment of hormone-responsive and stress-related elements in *BnaBAHD* promoters, while RNA-Seq profiles under nitrogen deficiency and phytohormone treatments demonstrated their broad involvement in abiotic stress responses. Notably, functional analyses confirmed that the seed-specific Clade V homologs *BnaBAHD040* and *BnaBAHD120* mediate the biosynthesis of structurally diverse *O*- and *N*-acylated metabolites critical for plant stress resilience. Our heterologous expression provides an experimental validation of the catalytic roles of *BnaBAHDs* in acylated metabolite biosynthesis, offering novel mechanistic insights into their contributions to plant secondary metabolism. Collectively, these findings not only confirm the functional role of *BnaBAHDs* in stress adaptation but also establish a theoretical framework for targeted genetic improvement of stress tolerance and yield-related traits in *B. napus*.

## Data Availability

All additional datasets supporting the findings of this study are included within the article and Supplementary Materials.

## Supplementary Materials

The following supporting information can be downloaded at: https://www.mdpi.com/article/10.3390/plants14142183/s1, Supplementary Figure S1: Functional characterization of *BnaBAHD120* through transient expression in *N. benthamiana*.; Table S1: Physicochemical properties of BAHD proteins in *Arabidopsis* and three *Brassica* Species.; Table S2: Motif sequences identified by MEME tools in *BnaBAHD* gene family.; Table S3: Identification of characteristic-conserved domains HXXXD and DFGWG in *BnaBAHD* gene family.; Table S4: Ka, Ks, and Ka/Ks analysis of *BAHD*-duplicated genes from *B. napus*.; Table S5: Ka, Ks, and Ka/Ks analysis of *BAHD*-duplicated genes between *B. oleracea* and *B. napus*.; Table S6: Ka, Ks, and Ka/Ks analysis of *BAHD*-duplicated genes between *B. rape* and *B. napus*.; Table S7: Transcriptome data of *BnaBAHD* family genes in various organs.; Table S8: qRT-PCR Primer used in this study.; Table S9: The detailed information and contents of the upregulated acylated metabolites.

## References

[1] D’Auria J.C. (2006). Acyltransferases in Plants: A Good Time to Be BAHD. Curr. Opin. Plant Biol..

[2] D’Auria J.C., Gershenzon J. (2005). The Secondary Metabolism of *Arabidopsis thaliana*: Growing like a Weed. Curr. Opin. Plant Biol..

[3] Pascal S., Bernard A., Sorel M., Pervent M., Vile D., Haslam R.P., Napier J.A., Lessire R., Domergue F., Joubès J. (2013). The *Arabidopsis Cer26* Mutant, like the *Cer26* Mutant, Is Specifically Affected in the Very Long Chain Fatty Acid Elongation Process. Plant J..

[4] Zhang W., Li J., Dong Y., Huang Y., Qi Y., Bai H., Li H., Shi L. (2024). Genome-Wide Identification and Expression of BAHD Acyltransferase Gene Family Shed Novel Insights into the Regulation of Linalyl Acetate and Lavandulyl Acetate in Lavender. J. Plant Physiol..

[5] Xu L., Zeisler V., Schreiber L., Gao J., Hu K., Wen J., Yi B., Shen J., Ma C., Tu J. (2017). Overexpression of the Novel Arabidopsis Gene *At5g02890* Alters Inflorescence Stem Wax Composition and Affects Phytohormone Homeostasis. Front. Plant Sci..

[6] Dudareva N., D’Auria J.C., Nam K.H., Raguso R.A., Pichersky E. (1998). Acetyl-CoA:Benzylalcohol Acetyltransferase—An Enzyme Involved in Floral Scent Production in *Clarkia breweri*. Plant J..

[7] Fujiwara H., Tanaka Y., Fukui Y., Nakao M., Ashikari T., Kusumi T. (1997). Anthocyanin 5-Aromatic Acyltransferase from *Gentiana triflora*. Purification, Characterization and Its Role in Anthocyanin Biosynthesis. Eur. J. Biochem..

[8] Yang Q., Reinhard K., Schiltz E., Matern U. (1997). Characterization and Heterologous Expression of Hydroxycinnamoyl/Benzoyl-CoA:Anthranilate N-Hydroxycinnamoyl/Benzoyltransferase from Elicited Cell Cultures of Carnation, *Dianthus caryophyllus* L.. Plant Mol. Biol..

[9] St-Pierre B., Laflamme P., Alarco A., De Luca V. (1998). The Terminal *O*-Acetyltransferase Involved in Vindoline Biosynthesis Defines a New Class of Proteins Responsible for Coenzyme A-Dependent Acyl Transfer. Plant J..

[10] Yu X.-H., Chen M.-H., Liu C.-J. (2008). Nucleocytoplasmic-Localized Acyltransferases Catalyze the Malonylation of 7-O-Glycosidic (Iso)Flavones in *Medicago truncatula*. Plant J..

[11] Ma X., Koepke J., Panjikar S., Fritzsch G., Stöckigt J. (2005). Crystal Structure of Vinorine Synthase, the First Representative of the BAHD Superfamily. J. Biol. Chem..

[12] Bontpart T., Cheynier V., Ageorges A., Terrier N. (2015). BAHD or SCPL Acyltransferase? What a Dilemma for Acylation in the World of Plant Phenolic Compounds. New Phytol..

[13] Molina I., Kosma D. (2015). Role of HXXXD-Motif/BAHD Acyltransferases in the Biosynthesis of Extracellular Lipids. Plant Cell Rep..

[14] Yu X.-H., Gou J.-Y., Liu C.-J. (2009). BAHD Superfamily of Acyl-CoA Dependent Acyltransferases in *Populus* and *Arabidopsis*: Bioinformatics and Gene Expression. Plant Mol. Biol..

[15] Xu D., Wang Z., Zhuang W., Zhang F., Xie Y., Wang T. (2024). Genome-Wide Identification and Expression Pattern Analysis of BAHD Acyltransferase Family in *Taxus mairei*. J. Mol. Sci..

[16] Aktar S., Bai P., Wang L., Xun H., Zhang R., Wu L., He M., Cheng H., Wang L., Wei K. (2022). Identification of a BAHD Acyltransferase Gene Involved in Plant Growth and Secondary Metabolism in Tea Plants. Plants.

[17] Yuan Z., Yang H., Pan L., Zhao W., Liang L., Gatera A., Tucker M.R., Xu D. (2022). Systematic Identification and Expression Profiles of the BAHD Superfamily Acyltransferases in Barley (*Hordeum vulgare*). Sci. Rep..

[18] De Vries L., MacKay H.A., Smith R.A., Mottiar Y., Karlen S.D., Unda F., Muirragui E., Bingman C., Vander Meulen K., Beebe E.T. (2022). pHBMT1, a BAHD-Family Monolignol Acyltransferase, Mediates Lignin Acylation in Poplar. Plant Physiol..

[19] Liu C., Qiao X., Li Q., Zeng W., Wei S., Wang X., Chen Y., Wu X., Wu J., Yin H. (2020). Genome-Wide Comparative Analysis of the BAHD Superfamily in Seven Rosaceae Species and Expression Analysis in Pear (*Pyrus bretschneideri*). BMC Plant Biol..

[20] Torrens-Spence M.P., Bobokalonova A., Carballo V., Glinkerman C.M., Pluskal T., Shen A., Weng J.-K. (2019). PBS3 and EPS1 Complete Salicylic Acid Biosynthesis from Isochorismate in Arabidopsis. Mol. Plant.

[21] Panikashvili D., Shi J.X., Schreiber L., Aharoni A. (2009). The Arabidopsis *DCR* Encoding a Soluble BAHD Acyltransferase Is Required for Cutin Polyester Formation and Seed Hydration Properties. Plant Physiol..

[22] Cumplido-Laso G., Medina-Puche L., Moyano E., Hoffmann T., Sinz Q., Ring L., Studart-Wittkowski C., Caballero J.L., Schwab W., Muñoz-Blanco J. (2012). The Fruit Ripening-Related Gene FaAAT2 Encodes an Acyl Transferase Involved in Strawberry Aroma Biogenesis. J. Exp. Bot..

[23] Li D., Xu Y., Xu G., Gu L., Li D., Shu H. (2006). Molecular Cloning and Expression of a Gene Encoding Alcohol Acyltransferase (MdAAT2) from Apple (Cv. Golden Delicious). Phytochemistry.

[24] Zhang B., Shen J., Wei W., Xi W., Xu C.-J., Ferguson I., Chen K. (2010). Expression of Genes Associated with Aroma Formation Derived from the Fatty Acid Pathway during Peach Fruit Ripening. J. Agric. Food Chem..

[25] Xu D., Wang Z., Zhuang W., Wang T., Xie Y. (2023). Family Characteristics, Phylogenetic Reconstruction, and Potential Applications of the Plant BAHD Acyltransferase Family. Front. Plant Sci..

[26] Wang C., Li J., Ma M., Lin Z., Hu W., Lin W., Zhang P. (2021). Structural and Biochemical Insights into Two BAHD Acyltransferases (*AtSHT* and *AtSDT*) Involved in Phenolamide Biosynthesis. Front. Plant Sci..

[27] Sonawane P.D., Gharat S.A., Jozwiak A., Barbole R., Heinicke S., Almekias-Siegl E., Meir S., Rogachev I., Connor S.E.O., Giri A.P. (2023). A BAHD-Type Acyltransferase Concludes the Biosynthetic Pathway of Non-Bitter Glycoalkaloids in Ripe Tomato Fruit. Nat. Commun..

[28] Tan Z., Han X., Dai C., Lu S., He H., Yao X., Chen P., Yang C., Zhao L., Yang Q. (2024). Functional Genomics of *Brassica napus*: Progress, Challenges, and Perspectives. J. Integr. Plant Biol..

[29] Zheng Q., Liu K. (2022). Worldwide Rapeseed (*Brassica napus* L.) Research: A Bibliometric Analysis during 2011–2021. Oil Crop Sci..

[30] Chalhoub B., Denoeud F., Liu S., Parkin I.A.P., Tang H., Wang X., Chiquet J., Belcram H., Tong C., Samans B. (2014). Early Allopolyploid Evolution in the Post-Neolithic *Brassica napus* Oilseed Genome. Science.

[31] Xia Y., Nikolau B.J., Schnable P.S. (1996). Cloning and Characterization of *CER2*, an *Arabidopsis* Gene That Affects Cuticular Wax Accumulation. Plant Cell.

[32] Wang M., Liu X., Wang R., Li W., Rodermel S., Yu F. (2012). Overexpression of a Putative *Arabidopsis* BAHD Acyltransferase Causes Dwarfism That Can Be Rescued by Brassinosteroid. J. Exp. Bot..

[33] Wang Z.-Y., Bai M.-Y., Oh E., Zhu J.-Y. (2012). Brassinosteroid Signaling Network and Regulation of Photomorphogenesis. Annu. Rev. Genet..

[34] Jeon H.S., Jang E., Kim J., Kim S.H., Lee M.-H., Nam M.H., Tobimatsu Y., Park O.K. (2023). Pathogen-Induced Autophagy Regulates Monolignol Transport and Lignin Formation in Plant Immunity. Autophagy.

[35] Zhang B., Sztojka B., Escamez S., Vanholme R., Hedenström M., Wang Y., Turumtay H., Gorzsás A., Boerjan W., Tuominen H. (2020). PIRIN2 Suppresses S-type Lignin Accumulation in a Noncell-autonomous Manner in Arabidopsis Xylem Elements. New Phytol..

[36] Simpson J.P., Kim C.Y., Kaur A., Weng J., Dilkes B., Chapple C. (2024). Genome-wide Association Identifies a BAHD Acyltransferase Activity That Assembles an Ester of Glucuronosylglycerol and Phenylacetic Acid. Plant J..

[37] Grienenberger E., Besseau S., Geoffroy P., Debayle D., Heintz D., Lapierre C., Pollet B., Heitz T., Legrand M. (2009). A BAHD Acyltransferase Is Expressed in the Tapetum of *Arabidopsis* Anthers and Is Involved in the Synthesis of Hydroxycinnamoyl Spermidines. Plant J..

[38] Leshem Y., Johnson C., Wuest S.E., Song X., Ngo Q.A., Grossniklaus U., Sundaresan V. (2012). Molecular Characterization of the *Glauce* Mutant: A Central Cell–Specific Function Is Required for Double Fertilization in *Arabidopsis*. Plant Cell.

[39] Gou J.-Y., Yu X.-H., Liu C.-J. (2009). A Hydroxycinnamoyltransferase Responsible for Synthesizing Suberin Aromatics in *Arabidopsis*. Proc. Natl. Acad. Sci. USA.

[40] Kosma D.K., Molina I., Ohlrogge J.B., Pollard M. (2012). Identification of an Arabidopsis Fatty Alcohol:Caffeoyl-Coenzyme A Acyltransferase Required for the Synthesis of Alkyl Hydroxycinnamates in Root Waxes. Plant Physiol..

[41] D’Auria J.C., Pichersky E., Schaub A., Hansel A., Gershenzon J. (2007). Characterization of a BAHD Acyltransferase Responsible for Producing the Green Leaf Volatile (*Z*)-3-hexen-1-yl Acetate in *Arabidopsis thaliana*. Plant J..

[42] Luo J., Fuell C., Parr A., Hill L., Bailey P., Elliott K., Fairhurst S.A., Martin C., Michael A.J. (2009). A Novel Polyamine Acyltransferase Responsible for the Accumulation of Spermidine Conjugates in *Arabidopsis* Seed. Plant Cell.

[43] D’Auria J.C., Reichelt M., Luck K., Svatoš A., Gershenzon J. (2007). Identification and Characterization of the BAHD Acyltransferase Malonyl CoA: Anthocyanidin 5-*O*-glucoside-6″-*O*-malonyltransferase (At5MAT) in *Arabidopsis thaliana*. FEBS Lett..

[44] Luo J., Nishiyama Y., Fuell C., Taguchi G., Elliott K., Hill L., Tanaka Y., Kitayama M., Yamazaki M., Bailey P. (2007). Convergent Evolution in the BAHD Family of Acyl Transferases: Identification and Characterization of Anthocyanin Acyl Transferases from *Arabidopsis thaliana*. Plant J..

[45] Sharma K., Hema K., Bhatraju N.K., Kukreti R., Das R.S., Gupta M.D., Syed M.A., Pasha M.A.Q. (2022). The Deleterious Impact of a Non-Synonymous SNP on Protein Structure and Function Is Apparent in Hypertension. J. Mol. Model..

[46] Oelschlaeger P. (2024). Molecular Mechanisms and the Significance of Synonymous Mutations. Biomolecules.

[47] Wang D., Zhang S., He F., Zhu J., Hu S., Yu J. (2009). How Do Variable Substitution Rates Influence Ka and Ks Calculations?. Genom. Proteom. Bioinform..

[48] Spoel S.H., Dong X. (2024). Salicylic Acid in Plant Immunity and Beyond. Plant Cell.

[49] Panche A.N., Diwan A.D., Chandra S.R. (2016). Flavonoids: An Overview. J. Nutr. Sci..

[50] Wasternack C., Strnad M. (2016). Jasmonate Signaling in Plant Stress Responses and Development—Active and Inactive Compounds. New Biotechnol..

[51] Moghe G., Kruse L.H., Petersen M., Scossa F., Fernie A.R., Gaquerel E., D’Auria J.C. (2023). BAHD Company: The Ever-Expanding Roles of the BAHD Acyltransferase Gene Family in Plants. Annu. Rev. Plant Biol..

[52] Qiao D., Yang C., Mi X., Tang M., Liang S., Chen Z. (2024). Genome-Wide Identification of Tea Plant (*Camellia sinensis*) BAHD Acyltransferases Reveals Their Role in Response to Herbivorous Pests. BMC Plant Biol..

[53] Song Z.-Z., Peng B., Gu Z.-X., Tang M.-L., Li B., Liang M.-X., Wang L.-M., Guo X.-T., Wang J.-P., Sha Y.-F. (2021). Site-Directed Mutagenesis Identified the Key Active Site Residues of Alcohol Acyltransferase PpAAT1 Responsible for Aroma Biosynthesis in Peach Fruits. Hortic. Res..

[54] Kruse L.H., Weigle A.T., Irfan M., Martínez-Gómez J., Chobirko J.D., Schaffer J.E., Bennett A.A., Specht C.D., Jez J.M., Shukla D. (2022). Orthology-Based Analysis Helps Map Evolutionary Diversification and Predict Substrate Class Use of BAHD Acyltransferases. Plant J..

[55] Li P., Xiao L., Du Q., Quan M., Song Y., He Y., Huang W., Xie J., Lv C., Wang D. (2023). Genomic Insights into Selection for Heterozygous Alleles and Woody Traits in *Populus tomentosa*. Plant Biotechnol. J..

[56] Tuominen L.K., Johnson V.E., Tsai C.-J. (2011). Differential Phylogenetic Expansions in BAHD Acyltransferases across Five Angiosperm Taxa and Evidence of Divergent Expression among *Populus* Paralogues. BMC Genom..

[57] Zhang Q., Guan P., Zhao L., Ma M., Xie L., Li Y., Zheng R., Ouyang W., Wang S., Li H. (2021). Asymmetric Epigenome Maps of Subgenomes Reveal Imbalanced Transcription and Distinct Evolutionary Trends in *Brassica napus*. Mol. Plant.

[58] Wang L., Chen K., Zhang M., Ye M., Qiao X. (2022). Catalytic Function, Mechanism, and Application of Plant Acyltransferases. Crit. Rev. Biotechnol..

[59] Kruse L.H., Fehr B., Chobirko J.D., Moghe G.D. (2023). Phylogenomic Analyses across Land Plants Reveals Motifs and Coexpression Patterns Useful for Functional Prediction in the BAHD Acyltransferase Family. Front. Plant Sci..

[60] Wang C., Chen C., Zhao X., Wu C., Kou X., Xue Z. (2022). Propyl Gallate Treatment Improves the Postharvest Quality of Winter Jujube (*Zizyphus jujuba* Mill. Cv. Dongzao) by Regulating Antioxidant Metabolism and Maintaining the Structure of Peel. Foods.

[61] Zhou J.-W., Ji P.-C., Wang C.-Y., Yang Y.-J., Zhao X.-Y., Tang H.-Z., Tang S.-R. (2023). Synergistic Effect of Propyl Gallate and Antibiotics against Biofilms of *Serratia marcescens* and *Erwinia carotovora* in Vitro. LWT.

[62] Dakora F.D., Matiru V.N., Kanu A.S. (2015). Rhizosphere Ecology of Lumichrome and Riboflavin, Two Bacterial Signal Molecules Eliciting Developmental Changes in Plants. Front. Plant Sci..

[63] Casida J.E. (1980). Pyrethrum Flowers and Pyrethroid Insecticides. Environ. Health Perspect..

[64] Ferroni C., Bassetti L., Borzatta V., Capparella E., Gobbi C., Guerrini A., Varchi G. (2015). Polyenylcyclopropane Carboxylic Esters with High Insecticidal Activity. Pest Manag. Sci..

[65] Chen H., Wang T., He X., Cai X., Lin R., Liang J., Wu J., King G., Wang X. (2022). BRAD V3.0: An Upgraded Brassicaceae Database. Nucleic Acids Res..

[66] Yang Z., Wang S., Wei L., Huang Y., Liu D., Jia Y., Luo C., Lin Y., Liang C., Hu Y. (2023). BnIR: A Multi-Omics Database with Various Tools for *Brassica napus* Research and Breeding. Mol. Plant.

[67] Mistry J., Chuguransky S., Williams L., Qureshi M., Salazar G.A., Sonnhammer E.L.L., Tosatto S.C.E., Paladin L., Raj S., Richardson L.J. (2021). Pfam: The Protein Families Database in 2021. Nucleic Acids Res..

[68] Altschul S. (1997). Gapped BLAST and PSI-BLAST: A New Generation of Protein Database Search Programs. Nucleic Acids Res..

[69] Wang J., Chitsaz F., Derbyshire M.K., Gonzales N.R., Gwadz M., Lu S., Marchler G.H., Song J.S., Thanki N., Yamashita R.A. (2023). The Conserved Domain Database in 2023. Nucleic Acids Res..

[70] Bailey T.L., Johnson J., Grant C.E., Noble W.S. (2015). The MEME Suite. Nucleic Acids Res..

[71] Duvaud S., Gabella C., Lisacek F., Stockinger H., Ioannidis V., Durinx C. (2021). Expasy, the Swiss Bioinformatics Resource Portal, as Designed by Its Users. Nucleic Acids Res..

[72] Letunic I., Bork P. (2024). Interactive Tree of Life (iTOL) v6: Recent Updates to the Phylogenetic Tree Display and Annotation Tool. Nucleic Acids Res..

[73] Chao J., Li Z., Sun Y., Aluko O.O., Wu X., Wang Q., Liu G. (2021). MG2C: A User-Friendly Online Tool for Drawing Genetic Maps. Mol. Hortic..

[74] Wang Y., Tang H., DeBarry J.D., Tan X., Li J., Wang X., Lee T.-h., Jin H., Marler B., Guo H. (2012). MCScanX: A Toolkit for Detection and Evolutionary Analysis of Gene Synteny and Collinearity. Nucleic Acids Res..

[75] Chen C., Wu Y., Xia R. (2022). A Painless Way to Customize Circos Plot: From Data Preparation to Visualization Using TBtools. iMeta.

[76] Lescot M. (2002). PlantCARE, a Database of Plant Cis-Acting Regulatory Elements and a Portal to Tools for in Silico Analysis of Promoter Sequences. Nucleic Acids Res..

[77] Chao H., Li T., Luo C., Huang H., Ruan Y., Li X., Niu Y., Fan Y., Sun W., Zhang K. (2020). BrassicaEDB: A Gene Expression Database for Brassica Crops. Int. J. Mol. Sci..

[78] Qu C., Zhu M., Hu R., Niu Y., Chen S., Zhao H., Li C., Wang Z., Yin N., Sun F. (2023). Comparative Genomic Analyses Reveal the Genetic Basis of the Yellow-Seed Trait in *Brassica napus*. Nat. Commun..

[79] Dong C., Qu G., Guo J., Wei F., Gao S., Sun Z., Jin L., Sun X., Rochaix J.-D., Miao Y. (2022). Rational Design of Geranylgeranyl Diphosphate Synthase Enhances Carotenoid Production and Improves Photosynthetic Efficiency in Nicotiana Tabacum. Sci. Bull..

[80] Wu G., Zhang L., Wu Y., Cao Y., Lu C. (2010). Comparison of Five Endogenous Reference Genes for Specific PCR Detection and Quantification of *Brassica napus*. J. Agric. Food Chem..

[81] Qu C., Yin N., Chen S., Wang S., Chen X., Zhao H., Shen S., Fu F., Zhou B., Xu X. (2020). Comparative Analysis of the Metabolic Profiles of Yellow- versus Black-Seeded Rapeseed Using UPLC-HESI-MS/MS and Transcriptome Analysis. J. Agric. Food Chem..

[82] Yin N.-W., Wang S.-X., Jia L.-D., Zhu M.-C., Yang J., Zhou B.-J., Yin J.-M., Lu K., Wang R., Li J.-N. (2019). Identification and Characterization of Major Constituents in Different-Colored Rapeseed Petals by UPLC-HESI-MS/MS. J. Agric. Food Chem..

[83] Tang Y., Zhang G., Jiang X., Shen S., Guan M., Tang Y., Sun F., Hu R., Chen S., Zhao H. (2023). Genome-Wide Association Study of Glucosinolate Metabolites (mGWAS) in *Brassica napus* L.. Plants.

[84] Shen S., Tang Y., Liu D., Chen L., Zhang Y., Ye K., Sun F., Wei X., Du H., Zhao H. (2025). Untargeted Metabolomics Analysis Reveals Differential Accumulation of Flavonoids between Yellow-Seeded and Black-Seeded Rapeseed Varieties. Plants.

[85] Tsugawa H., Ikeda K., Takahashi M., Satoh A., Mori Y., Uchino H., Okahashi N., Yamada Y., Tada I., Bonini P. (2020). A Lipidome Atlas in MS-DIAL 4. Nat. Biotechnol..

