# Supplementary material for: Genome-Wide Identification and Functional Characterization of the BAHD Acyltransferase Gene Family in Brassica napus L
Source: Plants (Basel). 2025 Jul 15;14(14):2183. doi: 10.3390/plants14142183 (PMC12299259; doi:10.3390/plants14142183)
Supplement: Supplementary file 1 [file plants-14-02183-s001.zip › Supplementary Figure.pdf]

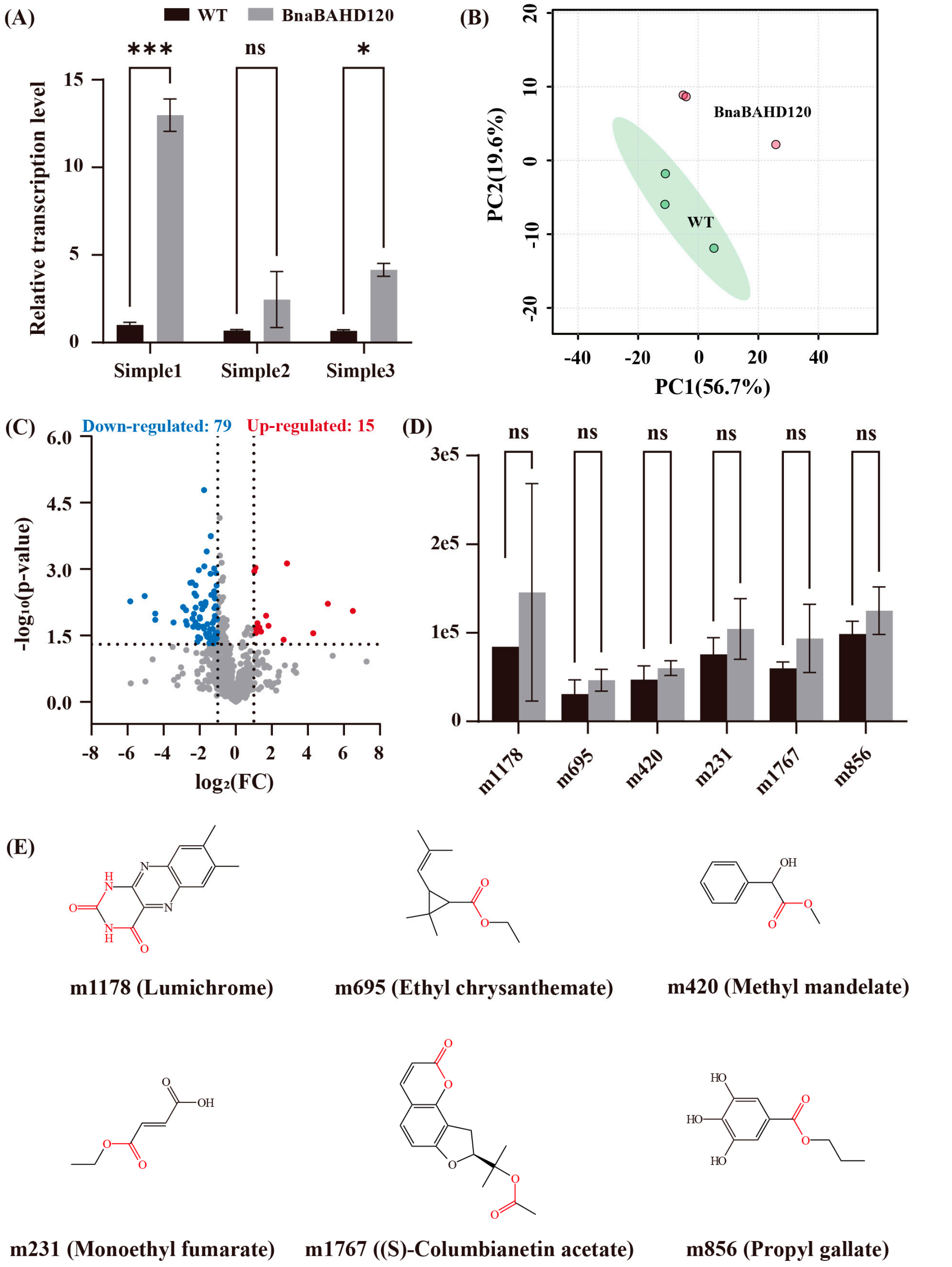

Supplementary Figure S1. Functional characterization of *BnaBAHD120* through transient expression in *N. benthamiana*. (A) qRT-PCR analysis of *BnaBAHD120* expression levels. (B) Principal component analysis (PCA) of metabolome data from *N. benthamiana* leaves. The first two principal components accounted for 76.3% of total variance (PC1: 56.7%, PC2: 19.6%). (C) Volcano plot analysis of differentially accumulated metabolites. The red points represent upregulated metabolites and blue represent downregulated metabolites. Dashed lines indicate significance thresholds. (D) Relative abundance of six up-regulated acylated metabolites. ns, not significant; *, $p < 0.05$; **, $p < 0.01$; ***, $p < 0.001$. (E) Chemical structures of

six up-regulated acylated metabolites. Ester (-COO-) and amide (-CONH-) bonds, the characteristic features of acylated metabolites, are highlighted in red.
